# Recent Advances in Biomedical Applications of Polymeric Nanoplatform Assisted with Two-Photon Absorption Process

**DOI:** 10.3390/polym14235134

**Published:** 2022-11-25

**Authors:** Subramaniyan Ramasundaram, Sivasangu Sobha, Gurusamy Saravanakumar, Tae Hwan Oh

**Affiliations:** 1 School of Chemical Engineering, Yeungnam University, Gyeongsan 38436, Republic of Korea; 2 OmniaMed Co., Ltd., Pohang 37666, Republic of Korea

**Keywords:** polymeric carrier, two-photon absorption, near infrared, fluorophore, drug delivery, imaging, photodynamic therapy, theranostic

## Abstract

Polymers are well-recognized carriers useful for delivering therapeutic drug and imaging probes to the target specified in the defined pathophysiological site. The functional drug molecules and imaging agents were chemically attached or physically loaded in the carrier polymer matrix via cleavable spacers. Using appropriate targeting moieties, these polymeric carriers (PCs) loaded with functional molecules were designed to realize target-specific delivery at the cellular level. The biodistribution of these carriers can be tracked using imaging agents with suitable imaging techniques. The drug molecules can be released by cleaving the spacers either by endogenous stimuli (e.g., pH, redox species, glucose level and enzymes) at the targeted physiological site or exogenous stimuli (e.g., light, electrical pulses, ultrasound and magnetism). Recently, two-photon absorption (2PA)-mediated drug delivery and imaging has gained significant attention because TPA from near-infrared light (700–950 nm, NIR) renders light energy similar to the one-photon absorption from ultraviolet (UV) light. NIR has been considered biologically safe unlike UV, which is harmful to soft tissues, cells and blood vessels. In addition to the heat and reactive oxygen species generating capability of 2PA molecules, 2PA-functionalized PCs were also found to be useful for treating diseases such as cancer by photothermal and photodynamic therapies. Herein, insights attained towards the design, synthesis and biomedical applications of 2PA-activated PCs are reviewed. In particular, specific focus is provided to the imaging and drug delivery applications with a special emphasis on multi-responsive platforms.

## 1. Introduction

The wide possibilities and realization witnessed in engineering a variety of structures and properties have made polymers a promising carrier matrix for functional biomedical applications such as drug delivery and imaging. The recent advances in modern polymerization techniques enables the synthesis of well-defined polymers with precise control over chemical composition, molecular weight, architecture and other functional properties such as stimuli-responsivity. Various strategies have advanced the biomedical applications of polymer-based drug delivery and imaging platforms, including chemical conjugation of active agents to the polymer scaffold, or physical encapsulation of active agents into self-assembled polymeric carriers (PCs) such as nanocapsules, micelles and polymersomes. Compared to free active agents, these PC-based systems endow several advantages, including improved solubility, long circulation and targeting [1,2,3]. Moreover, after exerting their functions, PCs based on biodegradable polymers can be easily degraded into non-toxic small molecules and exerted from the body [1]. A wide variety of natural and synthetic biodegradable polymers, such as polysaccharides, polyesters, polycarbonate, poly(amino acid), poly(aminoester), polyamides and polyurethane, have been used to design carriers for efficient and targeted delivery of active agents [4]. Over the past few decades, with appropriate design and fabrication, these PCs have found potential applications in the imaging and treatment of various diseases [5]. The specific advantages such as extravasation from the blood vessels and accumulation at tumor sites make nano-sized PCs a versatile and powerful platform for tumor-targeted drug delivery and imaging [6]. Recently, many PCs have also been explored for delivery of vaccines for coronaviruses (e.g., SARS-CoV, MERS-CoV, or hCoV). A small number of PC-based nanomedicines are under FDA approval, and several other are undergoing clinical trials and believed to be hopeful for future clinical use [7,8,9]. Remarkably, as they allow explicit spatial and temporal control of cargos at the desired sites, stimuli-responsive PCs have been emerging as on-demand drug delivery systems for effective therapy and precise imaging. 

Light as external medical stimulus is an easy and convenient tool useful for noninvasive therapy, image guided surgery, control over spatial resolution, localized polymerization and degradation of tissue engineering scaffolds [10,11]. When exposed to light, the photo-responsive PCs undergo chemical bond rupture and induce the dissociation of the carrier structure, thereby facilitating the release of encapsulated drugs. In some other cases, light exposure activates the imaging probes and photosensitizing agents for non-invasive imaging and photodynamic therapy (PDT), respectively [12,13]. Typically, PCs are designed to be responsive conventional one-photon absorption system using high-energy ultraviolet (UV) and visible (Vis.) radiations. The short wavelength (350–650 nm) of light use in the one-proton absorption system has the following shortcomings: limited penetration depth in tissues, photobleaching of probes meant for imaging, photodamaging of tissues and interference of autofluorescence of biological species, which are greatly limiting applications in the biomedical field. To surmount these issues, recently, photo-responsive PCs based on a two-photon absorption (2PA) system using low-energy near-infrared (NIR) radiation (700–950 nm) have been developed. When compared to UV and Vis radiations, NIR can deeply penetrate into tissues without any destruction or scattering [14,15]. Several studies have indicated that the depth of penetration of NIR radiation is largely dependent on the wavelength and also varies with type of tissue [16]. Henderson and Morries reported that about 0.45–2.90% of 810 nm light penetrated through 3 cm depth of brain tissue [17]. The use of a cheap continuous-wave laser with a low power density source, instead of a femtosecond pulse laser with high power density, was also found to be appealing for 2PA-based biomedical application using NIR radiation. Therefore, PCs responsive to 2PA possess tremendous potential in clinical medicine [18,19,20]. After a brief introduction of basic principle and relevance of 2PA in biomedical applications, this review will comprehensively summarize the recent advances in the two-photon-activated PCs for drug delivery, imaging, PDT and theranostic applications.

## 2. Basic Principle and Relevance of Two-Photon Absorption in Biomedical Applications

**Figure 1:**
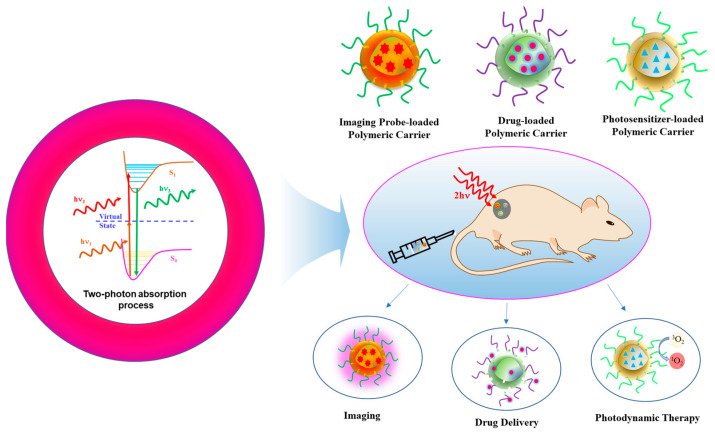
Basic principle of two-photon absorption process. Schematic representations of polymeric carriers loaded with imaging probe or therapeutic agents (drugs or photosensitizer) for two-photon-assisted imaging, drug delivery and photodynamic therapy.

In 1931, Maria Goppert-Mayer postulated the process of 2PA. Then, in 1961, 2PA was experimentally confirmed by Kaiser and Garret [21]. Figure 1 depicts the principle of 2PA and its biomedical applications. Constructive interference of light waves corresponding to two photons which are absorbed simultaneously by an ion, atom or molecule results in the promotion of electrons from ground state to excited state. The process by which two photons of the same frequency (degenerate 2PA) or different frequencies (non-degenerate) are absorbed by the molecular system proceeds through a stepwise process. In this process, the system first absorbs one photon and gets excited from the ground state (S_0_) to a temporary virtual state of higher energy. Before relaxing back to its initial state, the system immediately absorbs the second photon and gets excited to the real final electronic state (S_1_). When an excited electron returns to the ground state, the generation of a photon with energy greater than either two of the absorbed photons occurs. This phenomenon is known as 2PA [19,22,23]. The 2PA allows for the excitation of electrons in a molecule confined to volume of closer to the point of focus. Thus, pinpoint three-dimensional imaging can be realized by the polymeric nanoplatforms integrated with two-photon imaging probes [24,25]. A number of two-photon-responsive drug delivery systems that can accomplish the spatiotemporal control of drug accumulation at desired disease sites have also been developed to improve the therapeutic effect of the active agents. In these systems, a 2PA-generated photon induces the scission of incorporated photolabile groups within the carriers, resulting in the triggered release of drugs from the carriers to exert their therapeutic action [20]. Similarly, polymeric carriers encapsulated with photosensitizers (PS) that exhibit a large 2PA cross section have been utilized for two-photon excited photodynamic therapy. The irradiation of an NIR two-photon light source activates the PS to its excited state, which can subsequently interact with molecular oxygen to produce reactive oxygen species (ROS), leading to the destruction of the plasma membrane of tumor by localized oxidative stress. Notably, NIR radiation used in 2PA penetrates more deeply into tissues than the conventional one-photon process, where UV and visible light have been used. Though visible light is less harmful than UV, it can be scattered by tissues and absorbed strongly by blood. NIR is considered safer as it causes less or no damage to the normal tissues. Thus, 2PA-based systems are used to treat multidrug-resistant tumor cells by PDT alone or in combination with chemotherapy [3,26].

In the literature, in the biomedical field, 2PA is used for either one of the three applications mentioned previously or in a multifunction platform combining at least two applications and other methods of therapy such as chemotherapy. The recent development witnessed in each of the above applications of 2PA and its multifunctional platforms have been discussed in subsequent sections.

## 3. Polymeric Nanoplatforms for Two-Photon-Assisted Imaging

**Figure 2:**
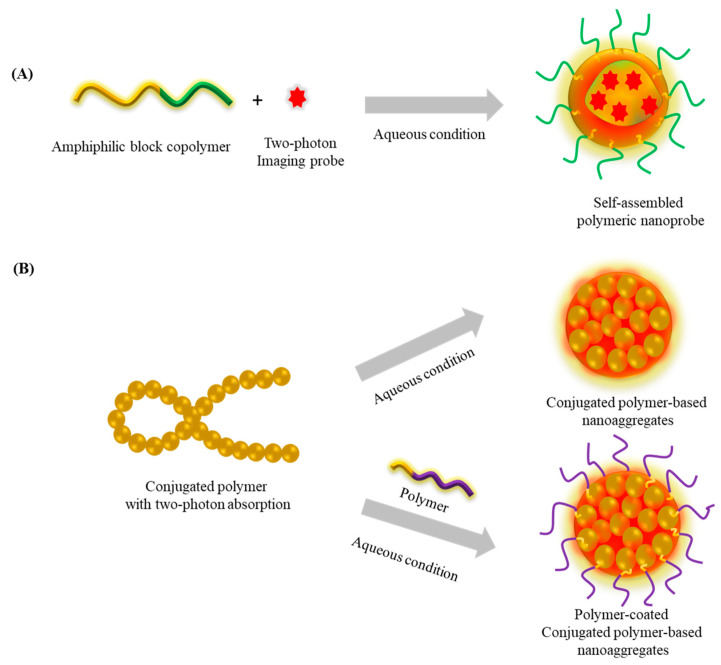
Schematic illustration of (**A**) two-photon imaging probe encapsulated polymeric nanoparticles and (**B**) conjugated polymer-based nanoaggregates with or without polymer coating for two-photon imaging.

PC-based two-photon imaging platforms have recently attracted significant interest due to their numerous advantages such as signal amplification, high photostability and low toxicity, compared with their small-molecule counterparts. There are two main strategies adopted in the development of PC-based systems for two-photon bioimaging. The first approach includes the physical encapsulation of small molecule two-photon imaging probes into the PCs, while the second one involves utilization of conjugated polymeric nanoassembly with large 2PA cross sections (Figure 2).

### 3.1. Small Molecule Probe-Loaded Systems

Given the excellent physical stability, facile fabrication and the opportunity to encapsulate multiple functional agents into the core along with the two-photon imaging probes, these physically loaded PCs have received significant attention towards both bioimaging and theranostic applications [27]. Although various conventional small molecule organic probes-loaded PCs have been prepared, the emission of these probes is often weakened in aggregates due to the phenomenon known as the aggregation-induced quenching (ACQ) effect. Increasing the loading content of these probes leads to reduced fluorescence and poor sensitivity. The discovery of fluorogens with aggregation-induced emission (AIE) addressed this ACQ issues [28]. Unlike traditionally fluorogenic probes, these AIE probes emit strong fluorescence in the aggregated state compared to the molecularly dissolved state. Although a number of possible mechanisms were hypothesized and proposed, the restriction of intramolecular motion has been widely considered the AIE working mechanism. Thus, encapsulating AIE probes within the core of polymeric nanoparticles could induce aggregation and suppress the intramolecular motion of the loaded probes, and the resulting nanoparticles can emit strong fluorescence and exhibit a relatively high photoluminescence quantum yield [29]. By taking advantage of this desired property, several AIE probe-loaded polymeric nanoparticles have been prepared for two-photon bioimaging. 

**Figure 3:**
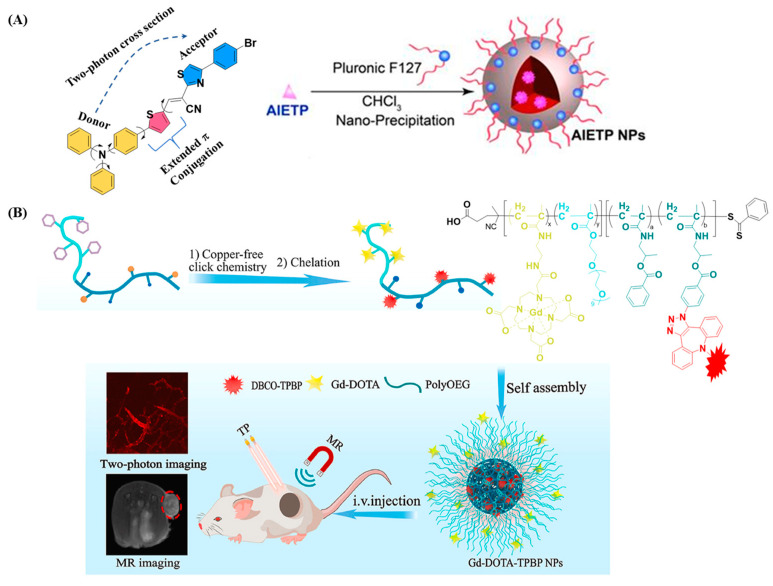
(**A**) Chemical structure of AIETP probe, and illustration for the preparation of AIETP-loaded Pluronic F127 nanoparticles. Adapted with permission from ref. [30] Copyright 2019 The Authors, Published by Theranostics. (**B**) Schematic representation of synthesis of amphiphilic block copolymer containing AIE probe TPBP and Gd-DOTA contrast agent for two-photon fluorescence and magnetic resonance imaging. Adopted with permission from ref. [44] Copyright 2022 The Authors. Published by KeAi Communications Co., Ltd.

Samanta et al. synthesized four acrylonitrile-based AIE-active two-photon fluorescence (AIETP) probes and encapsulated each into FDA-approved amphiphilic block copolymer Pluronic F127-based nanoparticles [30]. Then, by utilizing these four AIE probe-loaded nanoparticles, they comprehensively studied how the structural variations of the AIE probes influence the two-photon brain vascular imaging. Among the four synthesized AIE probes, the one with the phenyl-thiazole unit in the structure (AIETP, Figure 3A) facilitated the formation of excellent water-dispersible polymeric nanoparticles and showed improved photostability and a better 2PA cross section. In addition, this AIETP polymeric nanoparticle demonstrated two-photon NIR-II (1040 nm) excitability, which enables high-contrast vascular brain imaging with increased penetration depth. The NIR-II biological window (1000–1700 nm) is more promising for two-photon bioimaging because tissue components scatter and absorb less at the longer wavelengths, resulting in deeper penetration depth and higher spatial resolution, compared to the visible or conventional NIR-I window [31,32]. However, the design and synthesis of efficient fluorophores are crucial in NIR-II bioimaging. A crab-shaped donor-acceptor AIE active probe with a high quantum yield was synthesized and loaded into Pluronic F127 [33]. This polymer-loaded AIE nanoprobe showed high stability and a large two-photon absorption cross section (δ = 1.22 × 10^3^ GM). In vivo two-photon fluorescence imaging under NIR-II excitation (1300 nm) enables us to image 3D vascular information with high spatial resolution (sub 3.5 μm) and to visualize small blood vessels of ~5 μm as deep as 1065 μm in mouse brain. For two-photon fluorescence lifetime imaging (TP-FLIM), a thioxanthone-based AIE probe (TXO) was encapsulated within Pluronic F127 nanoparticles. As the TXO exhibits thermally activated delayed fluorescence properties and AIE features, the resulting polymeric nanoparticles exhibited ultralong fluorescence lifetime and efficient 2PA, making them useful for both in vitro and in vivo TP-FLIM [34]. Although the physical loading of AIE probes into a hydrophobic core of polymeric nanoparticles enhances its fluorescence intensity due to the spatial confinement that restricts intramolecular rotation of the probes, the chemical composition of the polymeric core also considerably influences the photophysical properties of AIE probes. In particular, AIE probes loaded into amphiphilic block copolymer nanoparticles with high hydrophobicity showed an improved fluorescence quantum yield compared to the one with less hydrophobicity. For example, owing to the increased hydrophobicity of poly(styrene) (PS) compared to that of poly(caprolactone) (PCL), the AIE probe loaded into poly(ethylene glycol)-*b*-PS (PEG-b-PS) copolymer nanoparticles exhibited higher fluorescence quantum yield compared to PEG-*b*-PCL [35]. Besides the polymeric core, engineering the surface of AIE probe-loaded polymeric nanoparticles also improved the fluorescence performance. When the shell of the AIE probe-loaded Pluronic F127 nanoparticles is coated with silica, their fluorescence quantum yield was greatly improved compared to the one without silica coating [36]. This enhanced characteristic of the silica shelled nanoparticles is ascribed to the non-polar microenvironment provided by the silica shell and reduced water and oxygen attack to the loaded AIE probes. Furthermore, encapsulating the AIE probe into folate conjugated poly([lactide-co-glycolide]-*b*-PEG (PLGA-*b*-PEG-Folate) nanoparticles showed potential for targeted cellular imaging [37]. In a similar way to small molecular fluorescent probe-loaded nanoparticles, several two-photon emissive polymers are also physically encapsulated into the PCs to improve optical stability and biocompatibility. Alifu et al. synthesized 2PA emissive polymer dots, encapsulated in poly(styrene-co-maleic anhydride) (PSMA) grafted with PEG matrix, and used them for imaging mouse ear and brain angiography [38]. When excited with 720–960 nm 2PA light, images with a penetration depth of 720 μm were obtained. For single-particle imaging in mouse brain, Khalin et al. prepared poly(methyl methacrylate)-sulfonate (PMMA-SO_3_H)-based nanoparticles loaded with probe octadecyl rhodamine B along with a bulky hydrophobic perfluorinated tetraphenylboronate as a fluorophore insulator to avoid the ACQ effect [39]. To impart stealth characteristics, these nanoparticles were further coated with pluronic F-127 and F-68. The as-prepared nanoparticles with 74 nm size and 20% loading content exhibited 150-fold higher single-particle brightness than 39 nm Fluospheres loaded with Nile Red. Through two-photon intravital microscopy, these stealth nanoparticles were able to track for 1 h in the vessels of mouse brain, while the nanoparticles without the stealth layer were readily eliminated. In particular, the dynamics of stealth nanoparticles can be tracked in real time in the microvasculature of living animals with subcellular resolution. Similarly, noninvasive and real-time two-photon imaging of thoracoepigastric veins of living balb/c nude mice has been realized by preparing PMMA-poly(methacrylic acid)-based micelles loaded with europium(Eu)-luminescent complexes with large 2PA cross sections at NIR photosensitization wavelengths [40]. Without the interference of autofluorescence from tissues, the spatiotemporal evolution of these nanoparticles in veins was observed for 2 h with 1–2 mm imaging depth and 80 μm lateral resolution.

In addition to the block copolymer nanoparticles, several polymeric lipid nanoparticles and polymer supramolecular nanoassemblies have also been employed for encapsulating two-photon small molecule imaging probes [41,42,43]. Recently, Shen et al. fabricated supramolecular NIR emission nanoparticles using a tetraphenylethene derivative with a methoxyl and vinyl pyridine salt-based AIE fluoroprobe, cucurbit [8] uril, and cancer cell targeting component hyaluronic acid modified cyclodextrin (*β*-CD) for cell targeting imaging. These nanoparticles showed less cytotoxicity when tested in A549 cells by with CCK-8 assay. These supramolecular nanoparticles solved the short wavelength excitation and emission problems of conventional supramolecular imaging systems and demonstrated deep tissue penetration and mitochondrial-targeted two-photon imaging [42].

Besides physical encapsulation, AIE-active small molecules are chemically conjugated to the amphiphilic copolymers and self-assembled into nanoprobe. Xiao et al. synthesized amphiphilic block copolymer covalently conjugated with a two-photon AIE probe (TPBP) and magnetic resonance contrast agent Gd-DOTA (Figure 3B) [44]. In aqueous condition, this copolymer formed self-assembled nanoparticles of about 20 nm, which can be circulated in blood for a long time. This polymeric nanoprobe provides an improved dual fluorescence and magnetic resonance imaging.

### 3.2. Conjugated Polymer-Based Systems

Conjugated polymers (CPs) consist of alternating σ and π-bonds on their polymer backbones, and thus, electrons can delocalize throughout the entire polymer chain, which produces interesting and specific photophysical and electrical properties. Compared to the traditional fluorescence materials, CPs show several distinguished features such as large extinction coefficient, high fluorescence efficiency and excellent photostability. In general, CPs are highly hydrophobic, and thus, to realize these materials for in vivo biomedical applications requires significant improvement of their aqueous solubility. In this direction, the following two strategies have been considered [45]. The first one is formulating CPs into nanoparticles (CPNs) by using nanoengineering techniques or integrating it into amphiphilic block copolymer nanoassemblies using appropriate methods, such as the physical encapsulation method discussed above. The second one is incorporating charged (anionic or cationic) or hydrophilic side chains to the CP hydrophobic backbone. Owing to advantages such as large 2PA cross section, high single-particle brightness, low cytotoxicity and facile surface modification, CPNs have been emerging as promising sensing platforms for both in vitro and in vivo two-photon bioimaging.

**Figure 4:**
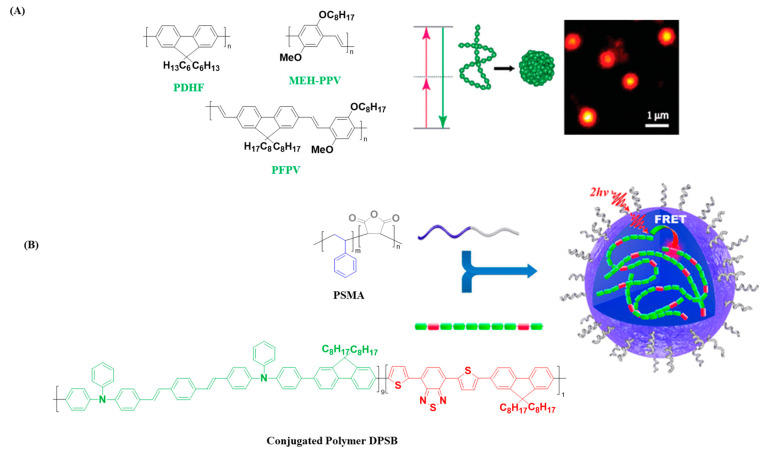
(**A**) Chemical structure of conjugated polymers (PDHF, MEH-PPV, and PFPV), and schematic illustration of formation of CNPs and fluorescence image of single PFPV particle immobilized on a glass coverslip obtained using two-photon excitation (800 nm). Adapted with permission from ref. [46]. Copyright 2007 American Chemical Society. (**B**) Chemical structures of conjugated polymer DPSB, amphiphilic block copolymer PSMA, and schematic illustration of DPSB-loaded CNPs. Adapted with permission from ref. [47] Copyright 2015 American Chemical Society.

McNeil and coworkers prepared CNPs from a series of CPs (polyfluorene derivative, polyfluorene copolymer and poly(phenylelenvinylene) derivative) by a reprecipitation method (Figure 4A) [46]. The two-photon action cross section of the as-prepared CNPs were found to be 3–4 orders of magnitude higher than the values of traditional fluorescence dyes, and an order of magnitude higher than those of inorganic quantum dots of comparable size. Single-particle fluorescence imaging was realized using relatively low laser power, demonstrating the potential of these CNPs for multiphoton fluorescence microscopy applications. In addition to the fabrications of CNPs using only CPs, the PC matrix encapsulated method has also been developed. Liu et al. prepared water dispersible CNPS by encapsulating red-emitting bis(diphenylaminostyryl)benzene (DPSB)-based conjugated polymers into the poly(styrene-co-maleic anhydride) (PSMA) matrix (Figure 4B) [47]. The size of CNPs was controlled by changing the feed ratio of DPSB to PSMA. By optimizing the feed ratio and initial solution concentration, a high quantum yield of 24% was obtained for the PSMA-CNPs and employed as a two-photon excitation nanoprobe for cell membrane imaging.

## 4. Polymeric Nanoplatforms for Two-Photon-Assisted Drug Delivery

Owing to the tunable physicochemical characteristics and facile incorporation of photo-responsive functional groups, PCs have attracted much interest in the field of light-responsive targeted drug delivery systems. In general, most of the photo-responsive moieties incorporated into PCs respond to UV or visible light, which has limited penetration depth and thus cannot induce photoresponsive changes to trigger drug release. However, as an exogeneous stimulus, two-photon light irradiation can penetrate more deeply into biological tissues and facilitate the precise spatiotemporal control of drug release.

### 4.1. 2-Diazo-1,2-Napthoquinone (DNQ)-Based Systems

**Figure 5:**
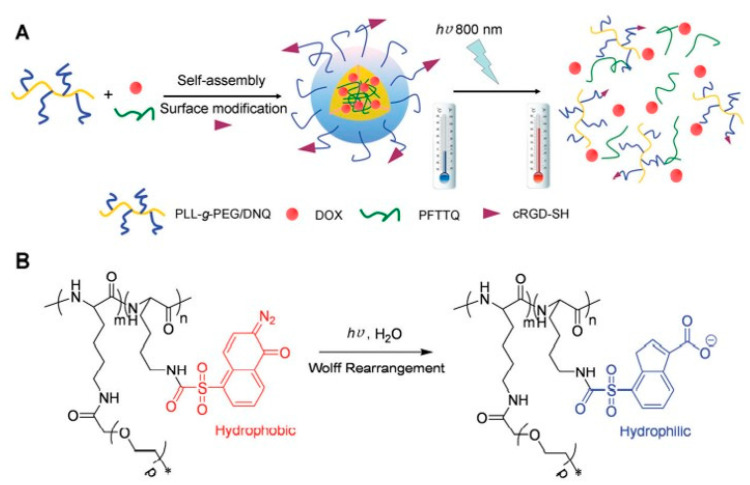
(**A**) Schematic illustration of preparation of PLL-g-PEG/DNQ nanoparticles and two-photon-mediated disassembly and concomitant drug release, and (**B**) chemical structure of PLL-g-PEG/DNQ and light-induced Wolff rearrangement. Adapted with permission from ref. [50] Copyright 2015 The Royal Society of Chemistry.

Among the various photoresponsive functional groups, DNQ is one of the most extensively studied units. This functional group induces the solubility switch and triggers drug release from the PCs. Goodwin et al. synthesized a functional PEG-lipid nanoparticle with 2-diazo-1,2-napthoquinones (DNQ) [48]. When this nanoparticle was irradiated with a 795 nm laser, the encapsulated Nile red dye was released from the nanoparticles through Wolff rearrangement of DNQ group via a two-photon process. These findings inspired many researchers to design two-photon responsive polymeric nanoparticles systems by judiciously incorporating DNQ moiety [49]. Yuan et al. developed a nanoplatform consisting of doxorubicin (DOX) and conjugated polymer (CP) meant for synchronous combination of photothermal therapy and chemotherapy (Figure 5A) [50]. The nanoparticle was formulated using an amphiphilic copolymer poly-L-lysine (PLL)-graft-PEG/DNQ (Figure 5B). A cyclic arginine-glycine-aspartic acid (cRGD) tripeptide was also attached to facilitate the accumulation of this nanosystem on cancer cells, where integrin αvβ3 gene overexpressed. When incubated with MDA-MB-231 cells and exposed to 800 nm laser irradiation for 20 min, DOX was released through disassembly of the nanoparticles by Wolff rearrangement induced conversion of DNQ moieties from hydrophobic to hydrophilic. Further, the release profile could be controlled by tuning the power density of the laser. The loaded CPs also endow in photothermal effect. Moreover, (IC50) of the combined chemotherapy and photothermal therapy (PTT) was 13.7 μg mL^−1^. The IC50 was 147.8 μg mL^−1^ for chemotherapy and 36.2 μg mL^−1^ for PTT. The combination index (C.I.) is 0.48 (<1), which indicates the synergistic effect for chemotherapy and PTT. By taking advantage of the DNQ group transformation, Sun et al. also constructed a two-photon-sensitive and sugar-targeted nanocarriers from degradable and dendritic amphiphiles [51].

### 4.2. Coumarin-Based Systems

The coumarinyl ester can be more easily cleaved by two-photon NIR light compared to the o-nitrobenzyl group. Hartner et al. studied the use of 2PA-induced cycloreversion of photodimerized 7-(*tert*-butyldimethylsilyloxy)-coumarin (TBS-coumarin) derivatives in both solution and incorporated into PMMA matrix [52]. When exposed to two-photon excitation, TBS-protected 7-hydroxycourmarin monomer was released from both conditions, indicating the potential of this transformation for two-photon controlled drug delivery applications. Along this line of research, they synthesized PMMA-based copolymer with photocleavable dicoumarin moieties and subsequently conjugated with an alkylating neoplastic drug molecule, chlorambucil [53]. As expected, when exposed to two-photon excitation with a 532 nm laser, the drug chlorambucil was released from the polymer matrix. Kim et al. conjugated a precures of the cataract-healing drug 5-florouracil (5-FU) to the backbone of polymers used for making intraocular lenses (IOL) via a coumarin linker [54]. The excitation of two-photon light (532 nm) triggered the release of 5-FU from the lens polymer matrix, which is useful for preventing the occurrence of secondary cataracts after implantation of IOL.

**Figure 6:**
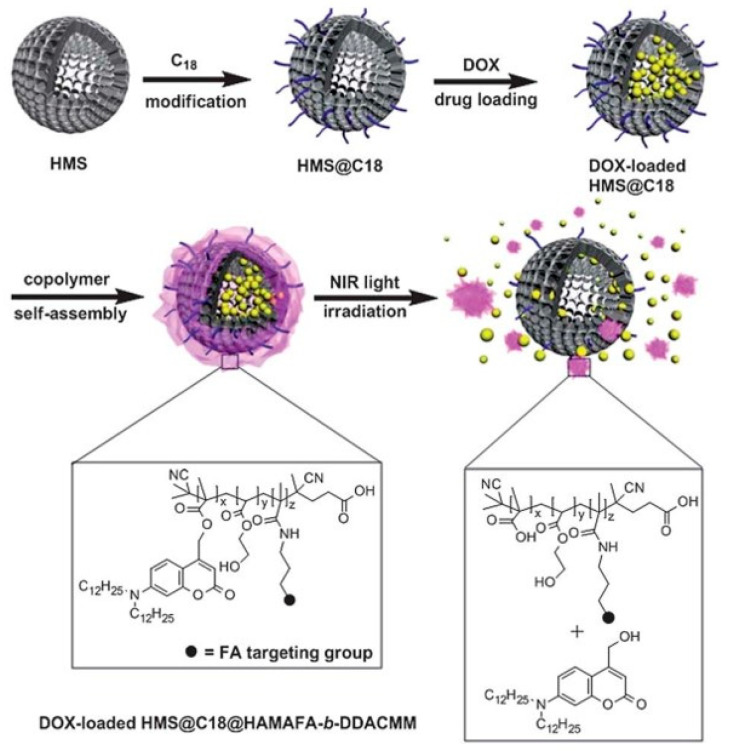
Schematic illustration of the preparation of HMS@C18@HAMAFA-*b*-DDACMM for drug delivery and controlled release by degradation upon NIR light exposure. The HAMAFA-*b*-DDACMM is a coumarin and folic acid conjugated copolymer, where the abbreviation HAMAFA denotes hydroxyethylacrylate and N-(3-aminopropyl) methacrylamide-conjugated folic acid segment, and DDACMM denotes 7-(didodecylamino) coumarin-4-yl] methyl methacrylate. Adapted with permission from ref. [55]. Copyright 2013 The Royal Society of Chemistry.

A hybrid coumarin-containing photo-responsive nanocomposite was prepared for NIR light-controlled drug release via a two-photon process [55]. This photo-responsive nanocomposite was fabricated by coating an NIR-light-responsive block copolymer containing photoactive coumarin moiety and tumor-targeting folic acid group onto the surface of DOX-loaded octadecyltrimethoxysilane (C18)-modified hollow mesoporous silica nanoparticles (HMS@C18) through self-assembly (Figure 6). The folic acid moiety conferred selective folate receptor-mediated endocytosis to the nanocomposites, which showed good selectivity to the folate receptor (FR(+)) KB cells compared to FR(-) A549 cancer cells. Because of the high 2PA cross section of the coumarin moiety, the copolymers were disrupted when exposed to NIR femtosecond laser (800 nm), leading to the triggered release of DOX over the targeted tumor cells. Greater than 50% of the DOX loaded on the nanocomposites was released upon NIR light irradiation.

### 4.3. o-Nitrobenzyl-Based Systems

Although the o-nitrobenzyl group is less sensitive to NIR light compared to UV light, this group can respond to NIR light via a two-photon absorption process. In response to two-photon irradiation, this group induced a polarity change and triggered the release of active agents. Since UV absorbers are used in IOL, they may diminish or suppress the two-photon-triggered drug release from the polymer. Kehrloesser et al. addressed this problem by choosing a suitable UV absorber that is compatible with 2PA and does not interfere with two-photon-mediated drug release [56]. They selected 2(4-benzoyl-3-hydroxy phenoxy) ethyl acrylate (BHP-EA) as the UV absorber and *o*-nitrobenzyl moiety as the photo-cleavable linker. IOLs were prepared using acrylic polymer with *o*-nitrobenzyl group carrying BHP-EA and the drug 5-FU. When evaluated for 2PA-triggered drug delivery, no photochemical degradation of the UV absorber was observed, and the release rate of 5-FU was not significantly affected.

### 4.4. Other Systems

Using a popular light-sensitive azobenzene moiety, Huang et al. reported supramolecular conjugated unimicelles for two-photon-triggered drug release [57]. These unimicelles were constructed by taking the advantages of host–guest interactions between a *β*-CD-grafted hyperbranched conjugated polymer and azobenzene-functionalized PEG. DOX was effectively loaded into these micelles with high loading efficiency. Upon NIR-light irradiation (800 nm), these micelles triggered the release of DOX in cancer cells through photoisomerization of azobenzene via a two-photon excited fluorescence energy transfer process, resulting in effective antitumor therapy. Oleincizak et al. synthesized a two-photon degradable copolymer containing photocleavable moieties on the backbone by polymerizing adipoyl chloride, 1,6-hexanediol and a light-sensitive monomer with high two-photon absorbing efficiency [58]. The model dye molecule Nile Red was readily released from this polymer nanoparticle through backbone degradation when exposed to NIR light radiation, demonstrating its potential in two-photon drug delivery.

## 5. Polymeric Nanoplatforms for Two-Photon-Assisted Gene Delivery

Apart from the delivery of small molecule drugs, multifunctional polymeric gene delivery carriers assisted with two-photon irradiation have been developed for the precise and effective treatment of both acquired and genetic diseases [10,59]. In recent years, polymer-based non-viral gene delivery systems are emerging as a potential alterative to viral-based systems because of the improved biocompatibility and minimal immunogenicity [4,60]. Among these, cationic polymers, which can easily form electrostatic complexes with anionic genetic materials including antisense oligodeoxynucelotides, small interfering RNA (siRNA) and other forms of nucleic acid, have been widely investigated as non-viral systems. Thus, semiconducting CPs bearing positive charges are also studied as non-viral gene carriers [61,62]. Although these cationic CPs can be complex negatively charged therapeutic nucleic acids and allow us to monitor the cellular internalization, their low charge density and lack of intracellular release mechanism severely limited gene transfection efficiency. To address this issue, two-photon-induced charge-variable conjugated polyelectrolyte brushes with high-density cationic charges and photodegradable *o*-nitrobenzyl groups on the side chain were prepared using poly(phenylene ethynylene)-based semiconducting polymer backbone, which also served as an upconversion agent [62]. The dense cationic charges enabled remarkable complex formation with siRNA. The large 2PA cross section of conjugated polyelectrolyte prompted two-photon-induced photolysis of photoresponsive side chains and facilitated charge transformation from cations to zwitterions, which triggered the efficient intracellular release of siRNA. In vitro studies demonstrate that these polyelectrolyte brushes can efficiently knock out of targeted Plk1 mRNA to 24.7% under 720 nm illumination. The positively charged CP-based carriers are also used for the delivery of plasmid DNA. The gene expression efficiency of CP-based systems was greatly improved compared to that of lipofectamine, a commonly used gene delivery vector [61].

Instead of utilizing positively charged CPs, few studies have conjugated two-photon active agents to cationic polyethyleneimine (PEI), another widely used gene carrier, for efficient two-photon-assisted gene delivery. For example, Hayek et al. labeled PEI (25kDa) with a bis-stilbenyl-based two-photon active dye molecule and employed for gene delivery and imaging [63]. The dye-labeled PEI complexes showed comparable transfection efficiency and cytotoxicity to those of unlabeled complexes. Moreover, the size of the complexes did not affect much when both labeled and unlabeled PEI was combined at a ratio of 1:3. In vitro two-photon imaging of HeLa cells demonstrated efficient internalization of the complexes by the cells and their accumulation in perinuclear compartments, particularly at the endosomes. In general, the molecular weight of PEI strongly correlates with its toxicity and transfection efficiency. It has been reported that low molecular weight PEI (2kDa) modified with hydrophobic moieties could exert high gene transfection efficiency and good biocompatibility [64]. Thus, amphiphilic alkyl-PEG(2k) was synthesized and coated on the surface of photonic carbon dots (Cdots), and the resulting PEI-Cdots were evaluated as a carrier for the delivery of siRNA and DNA. The results demonstrated effective in vitro and in vivo gene transfection of PEI-Cdots. Moreover, the photonic Cdot endows two-photon imaging capabilities.

## 6. Polymeric Nanoplatforms for Two-Photon-Assisted Photodynamic Therapy

PDT is a minimally invasive treatment approved for certain cancers and non-cancerous disease states. It involves the administration of a photosensitizer, which is activated by light at specific wavelength, and generates cytotoxic species in the presence of oxygen.

**Figure 7:**
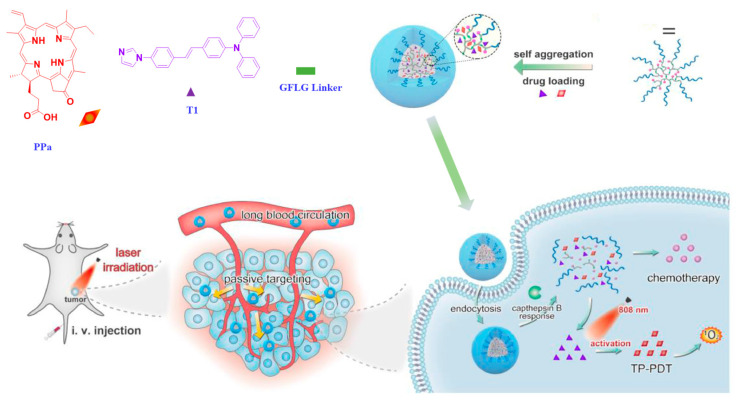
Preparation of cathepsin B-responsive dendritic prodrug and dendritic-[(GFLG-polyPTX)-block-polyOEGMA]. The self-assembled dendritic prodrug is loaded with TPA and PPa, leading to synergistic effect of chemotherapy and two-photon photodynamic therapy for breast tumor. Adapted with permission from ref. [66] Copyright 2021 Elsevier Ltd.

Luo et al. prepared a thermosensitive nanocomposite hydrogel from methoxy poly(ethylene glycol)-polylactide copolymer (mPEG-PDLLA) and Pluronic F127, which is approved for clinical use [65]. A two-photon active imidazole derivative with large TPA cross section in the NIR region and a second-generation photosensitizer pyropheophorbide a (PPa) were loaded into these mPEG-PDLLA micelles. Upon injection, at body temperature, these micelles were transformed into hydrogel and displayed a long-term retention within the tumor. This composite hydrogel was tested using 4T1 murine breast cancer bearing BALB/c mice. When the thermosensitive hydrogel is activated using NIR, a significant amount of ROS was generated in deep tissue and inhibited the growth of tumor. Further, when the thermosensitive hydrogel-treated cells were observed through two-photon confocal microscope, obituary morphology and bubbling were noticed in the regions of cell membrane and nucleus, which is the indication for the occurrence of PDT. In another study, Luo et al. encapsulated PPa and a two-photon active imidazole derivative into dendritic prodrug-based nanoparticles obtained using poly[oligo(ethylene glycol)methyl ether methacrylate]-functionalized dendritic paclitaxel (PTX) (Figure 7) [66]. The branches of the dendritic polymers and PTX were conjugated to the polymer via a short peptide Gly-Phe-Leu- Gly that was sensitive to cathepsin B, a lysosomal cysteine protease overexpressed in tumor cells. This peptide linkage protected the drug from undesired premature leakage during circulation in the blood. After passive accumulation at the tumor site, the drug was released in response to the tumor microenvironment. The efficacy of this polymeric prodrug-based nanoparticle was evaluated using BALB/c mice bearing 4T1 murine breast cancer. The chemo/two-photon combined nanosystems showed a much higher tumor growth inhibition (67.23%) compared to that of the group treated with PTX and PPa (48.92%). Further, this nanosystem also allowed for the tracking of drug localization by two-photon bioimaging.

Kandoth et al. developed a three-component self-assembled polymeric nanosystem comprising an epichlorohydrin-*β*-CD copolymer as the carrier, a zinc phthalocyanine as a photosensitizer and a tailored nitroaniline derivative as a nitric oxide photodonor [67]. When encapsulated into the nanosystems, these two photoresponsive guest molecules did not interfere with each other, which was confirmed using steady-state and time-resolved spectroscopic and photochemical techniques. Thus, each of these molecules can be operated in parallel under the light stimuli. The tissue distribution of the system can be imaged using the two-photon fluorescence microscopy. When irradiated with visible light, this nanosystem simultaneously triggered generation of cytotoxic species ^1^O_2_ and NO and resulted in death of carcinoma cells.

## 7. Polymeric Nanoplatforms for Two-Photon-Assisted Theranostic Applications

As discussed in the previous sections, PCs equipped with the 2PA probe enable imaging and drug delivery capabilities and PDT. Recently, significant efforts have been devoted to integrating the above modalities into one single theranostic platform. These integrated systems may allow for a safe, efficient and minimally invasive tool for personalized medicine. In this section, we will discuss a few representative theranostic polymeric systems for two-photon bioimaging, chemotherapy, PTT and PDT.

### 7.1. Cancer Theranostics

To date, polymeric nanoparticles are widely studied for cancer theranostics. Wang et al. prepared hybrid nanogels by immobilizing graphitic carbon dots (GCDs) within the PC matrix comprising a crosslinked PEG network and interpenetrated chitosan chains via an aqueous one-pot surfactant-free precipitation polymerization [68]. The biocompatible GCDs not only served as multifunctional confocal and two-photon imaging agent and pH-sensing probe, but also enhanced the loading capacity of hydrophobic anticancer drug, DOX, via π stacking mechanism. Due to their ability to absorb NIR and upconverting photoluminescent properties, GCDs provided the nanogels with high photothermal conversion ability and two-photon imaging ability. While the chitosan-induced swelling/deswelling regulated the drug release over the physiologically important pH range of 5.0–7.4, the thermoresponsive PEG network of the nanogels promoted the drug release through the local heat produced by GCDs under NIR irradiation. These results show the combined synergistic effect of the hybrid nanogels for treating cancer by chemo-photothermal modalities, as well for diagnostic imaging. Ardekani et al. synthesized photoresponsive nanoparticles based on the nitrogen-doped surface passivated (PEG, MW~200) carbon nanodots (CND-P) and anticancer drug DOX [69]. The nitrogen doping and surface passivation greatly improved their properties. For example, the quantum yield of CND-P was enhanced compared to the undoped CND and non-surface passivated CND by factors of 12.6 and 4.4, respectively. Under similar test conditions, the up-converted emission intensity of CND-P was increased by a factor of 4.5 compared to the non-surface passivated one. The CND-P was able to transport and release DOX into MCF-7 cancer cell through two-photon excitation (780 nm and 35 mW). The two-photon bioimaging indicated the internalization of CND-P in cytoplasm, nucleolus and nuclei of the MCF-7 cells. Due to the heat generation capability of CND-P, the DOX-mediated apoptosis of MCF-7 cells was increased through the combined chemo and photothermal effect.

**Figure 8:**
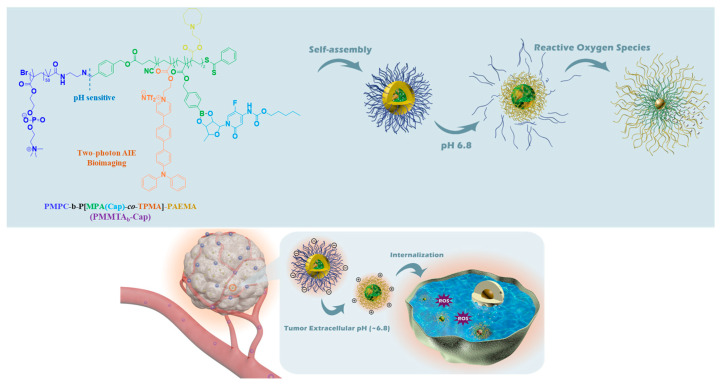
Schematic illustration for preparation of polymeric prodrug PMMTAb-Cap micelles with two-photon bioimaging, pH-triggered size shrinkage and charge-reversal, and ROS-triggered drug release. Adapted with permission from ref. [70]. Copyright 2019 American Chemical Society.

Y. Wang and co-workers developed several polymeric systems that are capable of simultaneous two-photon imaging and stimuli-responsive drug delivery. By employing two-photon AIE-based imaging probe and judiciously placing stimuli-responsive units within the amphiphilic block copolymers either at the main backbone or side chain, they prepared number of theranostic polymeric platforms for enhanced two-photon imaging and effective therapy. Using the combination of RAFT polymerization and chemical conjugation methods, they synthesized a prodrug block copolymer featuring a two-photon AIE fluorophore and an anticancer drug capecitabine (Cap), which has been applied for the treatment of multiple malignant tumors, alone or in combination regimens [70]. In brief, first an amine-end functionalized hydrophilic poly(2-methacryloyloxyethyl phosphorylcholine) (PMPC-NH_2_) was linked to an aldehyde-end functionalized poly(methacryloyl phenylboronic acid pinacol ester-*co*-TPMA)-*b*-poly(2-azepane ethylmethacrylate) (CHO-P(MPA-*co*-TPMA)-*b*-PAEMA) to introduce an acid-labile benzoyl imide bond on the main backbone. Second, the drug Cap was conjugated to MPA unit via ROS-responsive phenylboronate ester bond to obtain the final amphiphilic prodrug copolymer (PMMTA_b_-Cap), which can form self-assembled micelles under aqueous conditions (Figure 8). The TPMA unit in the copolymer is a two-photon fluorophore. The PAEMA segment is a charge-convertible unit that can become electropositive and induce hydrophobic to hydrophilic solubility switch under mild acidic (pH 6.8) tumor microenvironment. Due to AIE effect of the TPMA, these prodrug micelles exhibited two-photon imaging capability and were able to obtain deep tissue imaging with a depth of 150 µm. After accumulation at the acidic tumor tissue, the outer shell PMPC can be detached and decrease the size of the micelles while the inner PAEMA become hydrophilic and electropositive, leading to effective internalization of the micelles into the tumor cells. After cellular uptake, the elevated intracellular ROS can disrupt the boronic ester bond and trigger the release of Cap for effective tumor therapy. In a subsequent study, they have also reported another prodrug micellar system with a neutral hydrophilic pH-detachable PEG shell and a dimethylmaleic anhydride-grafted-polyethyleneimine segment charge reversable segment [71]. While the AIE two-photon probe was chemically conjugated to the hydrophobic chain end, the therapeutic drug DOX was conjugated to the main backbone via an acid-labile imine bond. When incubated with 4T1 cells, these micelles produced fluorescence image with good quality, in response to excitations with one photon (405 nm) and two-photon (800 nm) light irradiations. The in vivo potential of these micelles was investigated using BALB/c mice bearing 4T1 breast cancer tumors. Under 800 two-photon excitation, bright fluorescence signals were observed in hepatic and nephric tissues, and the images were visualized even at the depth of 150 µm, whereas the florescence signals under one-photon excitation rapidly declined with increased in scanning depth. After accumulation at the tumor tissues by the enhanced permeation and retention effect, the detachment of the PEG shell enhanced the uptake by charge reversal and subsequently released the DOX, leading to improved antitumor efficacy. Along this line of research, redox-responsive theranostic prodrug micelles were also prepared that were capable of releasing anticancer drug gemcitabine under elevated glutathione (GSH) [72]. As the concentration of the GSH is at least 4 times higher in some tumors compared to the normal cells, these micelles demonstrated good in vivo tumor-suppression ability. In another study, oxidation-responsive PEG-*b*-poly(L-glutamic acid)-selenide micelles equipped with AIE-active two-photon probe were prepared and physically loaded with curcumin (Cur), a well-known herbal compound that exerts several anticancer effects in various types of cancers [73]. These micelles also showed good deep two-photon tissue imaging. Under the oxidative environments of the tumor, these micelles were readily dissociated and released the drug Cur. The prolonged circulation and efficient accumulation of these micelles at the tumor tissue greatly improved their tumor inhibition ability. Similarly, the anticancer drug DOX was physically loaded into redox-responsive theranostic micelles with two-photon imaging capability, which can induce charge conversion under acidic condition and detach PEG shell and concomitant DOX release in response to high concentration of GSH in the tumor cells [74]. In vivo, ex vivo imaging and in vivo pharmacokinetic study demonstrated the potential of these micelles for deep-tissue imaging and cancer therapy. 

### 7.2. Atherosclerosis Theranostics

**Figure 9:**
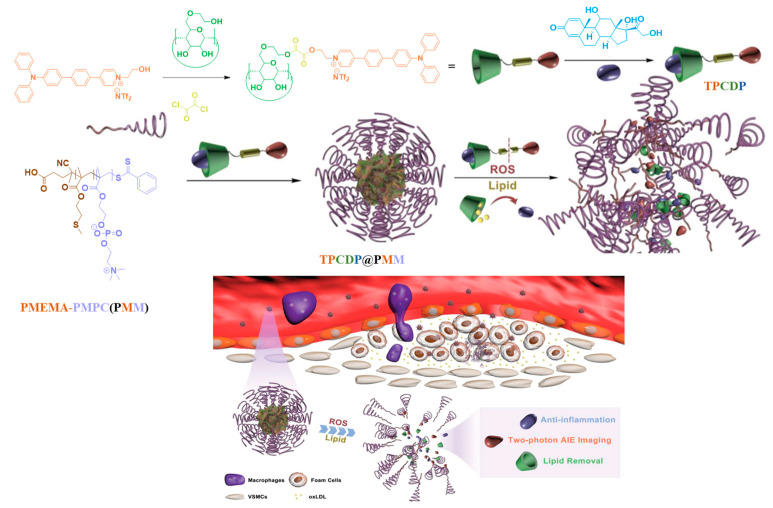
Schematic illustration of TPCDP@PMM theranostic micelles preparation and its responsive behaviors under overexpressed ROS and rich lipid. The TPCDP@PMM is designed to realize ROS-triggered micellar dissociation, rich lipid mediated drug Pred release and two-photon AIE imaging, which result in anti-tumor activity, lipid removal and clear dimensional two-photon imaging of atherosclerosis plaques. Adapted with permission from ref. [75]. Copyright 2020, Wiley-VCH GmbH.

Atherosclerosis is the gradual narrowing of the arteries caused by the buildup of plaque in the inner lining of an artery. It is one of the leading causes of death worldwide. The rupture of vulnerable atherosclerosis plaque is the major cause of sudden deaths. Therefore, imaging the structural and anatomic features of atherosclerotic plaque is crucial to inhibit the vulnerable atherosclerotic plaque progress. Recently, Ma et al. prepared supramolelcular theranostic micelles for combined two-photon AIE imaging of atherosclerosis diagnosis and two-pronged therapy [75]. These micelles are prepared by following the three steps. First, a two-photon fluorophore (TP) was covalently linked to *β*-CD via a ROS-responsive peroxalate ester to obtain a conjugate TPCD. Second, prednisolone (PRDL), a poorly soluble anti-inflammatory glucocorticoid, was encapsulated into *β*-CD via supramolecular interaction (Figure 9). Finally, the PRDL-loaded TPCD (TPCDP) was packed inside an amphiphilic poly(2-methyltho ethanol methacrylate)-*b*-PMPC (PMM) to form the theranostic micelles (TPCDP@PMM). The outer polymeric PMM shell can improve in vivo blood circulation and provide a chance for the accumulation of these micelles at the atherosclerotic lesion through the damaged vascular endothelium. The in vivo two-photon bioimaging of TPCD@PMMA on atherosclerotic ApoE-/- mice exhibited stronger fluorescence and more plaques were recognized. This confirmed the continuous accumulation of micelles. After accumulation, the overexpressed ROS level at the inflammatory tissue triggers a hydrophobic-to-hydrophilic switch of the PMEMA segment of the copolymer, leading to the disassembly of the structure. This process exposes the TPCDP to a high level of ROS and disrupts the peroxalate ester bond and facilitates the release of PRDL as well as the *β*-CD, resulting in two-pronged therapy via anti-inflammatory activity of PRDL and lipid removal feature of *β*-CD for atherosclerosis inhibition. These results suggest that these micelles can be promising nanoplatform for atherosclerosis theranostics. By employing a polymeric *β*-CD conjugate and a nitric oxide (NO) photodonor, a multifunctional biocompatible nanoconstruct has been synthesized for photo-controlled NO release with two-photon fluorescence imaging [76]. After synthesizing the polymeric *β*-CD conjugate using *β*-CD with epichlorohydrin under alkaline conditions, the NO photodonor with anthracene and nitroaniline structural unit was loaded into the polymeric conjugate to obtain the nanoconstruct. The NO release from the nanoconstruct was triggered by two-photon excitation using NIR laser (700 nm). The uncaged fluorescent co-product acted as a two-photon emission fluorescence reporter for the concomitant NO release. The release of NO in human squamous carcinoma cells was monitored by two-photon NIR fluorescence microscopy, which indicated the localization of fluorescent reporter in the cytoplasm. This nanoconstruct has potential for combined photo-activated therapies and bioimaging.

## 8. Conclusions and Outlook

The capability of excitation light utilized in 2PA on penetrating deep tissue without damaging normal tissue is providing impetus for the development of two-photon-based systems for biomedical applications. By integrating diverse modularly designed copolymers and two-photon AIE probe or conjugated polymers with large 2PA cross section, several polymeric nanoprobes have been developed for imaging various diseases. In most of the cases the well-resolved images were obtained with a tissue penetration depth of 150 µm. With appropriately designed two-photon probe with a large two-photon absorption cross section, under NIR-II excitation, small blood vessels as deep as 1064 μm in mouse brain are visualized. In recent years, 2PA-based bioimaging has been combined with nanoplatforms that perform smart drug delivery and ROS-mediated PDT. In most cases, NIR radiation of 800 nm was used as the 2PA excitation light. The combination of 2PA-based PDT and stimuli-responsive chemotherapeutic drugs have rendered effective cancer treatment. The photo-responsive functional moieties such as DNQ, coumarin and *o*-nitrobenzyl were carefully placed within the polymeric nanoparticles to trigger on-demand photo-regulated drug release for enhanced therapy. Most of these systems are limited to cancer therapy. Thus, more two-photon-based systems should be designed and evaluated for other diseases. Despite the potential benefits and numerous advantages of these two-photon-activated polymeric systems, a number of studies conducted were mostly limited to in vivo animal models. The findings from animal studies are often difficult to transfer to humans. Therefore, studies related to preclinical efficacy and safety in humans are needed. Nonetheless, it was evident from the literature that 2PA responsive bioimaging can be effectively combined with other stimuli-responsive drug delivery and therapeutic applications. Additionally, it is certain that, in the future, the prime focus of 2PA-based research will move towards the development of a multifunctional theranostic system capable of performing imaging, drug delivery and ROS-induced therapy.

## Data Availability

Upon reasonable request, the data supporting this investigation are available from the corresponding authors.

## References

[1] Mitchell M.J., Billingsley M.M., Haley R.M., Wechsler M.E., Peppas N.A., Langer R. (2021). Engineering precision nanoparticles for drug delivery. Nat. Rev. Drug Discov..

[2] Li J., Yu F., Chen Y., Oupický D. (2015). Polymeric drugs: Advances in the development of pharmacologically active polymers. J. Control. Release.

[3] Das S.S., Bharadwaj P., Bilal M., Barani M., Rahdar A., Taboada P., Bungau S., Kyzas G.Z. (2020). Stimuli-Responsive Polymeric Nanocarriers for Drug Delivery, Imaging, and Theragnosis. Polymers.

[4] Sung Y.K., Kim S.W. (2019). Recent advances in the development of gene delivery systems. Biomater. Res..

[5] Ali I., Alsehli M., Scotti L., Tullius Scotti M., Tsai S.-T., Yu R.-S., Hsieh M.F., Chen J.-C. (2020). Progress in Polymeric Nano-Medicines for Theranostic Cancer Treatment. Polymers.

[6] Khan M.I., Hossain M.I., Hossain M.K., Rubel M.H.K., Hossain K.M., Mahfuz A.M.U.B., Anik M.I. (2022). Recent Progress in Nanostructured Smart Drug Delivery Systems for Cancer Therapy: A Review. ACS Appl. Bio Mater..

[7] Vu M.N., Kelly H.G., Kent S.J., Wheatley A.K. (2021). Current and future nanoparticle vaccines for COVID-19. eBioMedicine.

[8] Park H., Otte A., Park K. (2022). Evolution of drug delivery systems: From 1950 to 2020 and beyond. J. Control. Release.

[9] Zhu M., Whittaker A.K., Han F.Y., Smith M.T. (2022). Journey to the Market: The Evolution of Biodegradable Drug Delivery Systems. Appl. Sci..

[10] Zhou Y., Ye H., Chen Y., Zhu R., Yin L. (2018). Photoresponsive Drug/Gene Delivery Systems. Biomacromolecules.

[11] Azagarsamy M.A., Anseth K.S. (2013). Wavelength-Controlled Photocleavage for the Orthogonal and Sequential Release of Multiple Proteins. Angew. Chem. Int. Ed..

[12] Sanchis A., Salvador J.P., Marco M.P. (2019). Light-induced mechanisms for nanocarrier’s cargo release. Colloids Surf. B. Biointerfaces.

[13] Linsley C.S., Wu B.M. (2017). Recent advances in light-responsive on-demand drug-delivery systems. Ther. Deliv..

[14] Hudson D.E., Hudson D.O., Wininger J.M., Richardson B.D. (2013). Penetration of Laser Light at 808 and 980 nm in Bovine Tissue Samples. Photomed. Laser Surg..

[15] Chen G., Shen J., Ohulchanskyy T.Y., Patel N.J., Kutikov A., Li Z., Song J., Pandey R.K., Ågren H., Prasad P.N. (2012). (α-NaYbF4:Tm3+)/CaF2 Core/Shell Nanoparticles with Efficient Near-Infrared to Near-Infrared Upconversion for High-Contrast Deep Tissue Bioimaging. ACS Nano.

[16] Padalkar M.V., Pleshko N. (2015). Wavelength-dependent penetration depth of near infrared radiation into cartilage. Analyst.

[17] Henderson T.A., Morries L.D. (2015). Near-infrared photonic energy penetration: Can infrared phototherapy effectively reach the human brain?. Neuropsychiatr. Dis. Treat..

[18] Liu G., Liu W., Dong C.-M. (2013). UV- and NIR-responsive polymeric nanomedicines for on-demand drug delivery. Polym. Chem..

[19] Zhu X., Su Q., Feng W., Li F. (2017). Anti-Stokes shift luminescent materials for bio-applications. Chem. Soc. Rev..

[20] Yang G., Liu J., Wu Y., Feng L., Liu Z. (2016). Near-infrared-light responsive nanoscale drug delivery systems for cancer treatment. Coord. Chem. Rev..

[21] Pawlicki M., Collins H.A., Denning R.G., Anderson H.L. (2009). Two-Photon Absorption and the Design of Two-Photon Dyes. Angew. Chem. Int. Ed..

[22] Bort G., Gallavardin T., Ogden D., Dalko P.I. (2013). From One-Photon to Two-Photon Probes: “Caged” Compounds, Actuators, and Photoswitches. Angew. Chem. Int. Ed..

[23] Alam M.M., Chattopadhyaya M., Chakrabarti S., Ruud K. (2014). Chemical Control of Channel Interference in Two-Photon Absorption Processes. Acc. Chem. Res..

[24] Potter S.M. (1996). Vital imaging: Two photons are better than one. Curr. Biol..

[25] Wu L., Liu J., Li P., Tang B., James T.D. (2021). Two-photon small-molecule fluorescence-based agents for sensing, imaging, and therapy within biological systems. Chem. Soc. Rev..

[26] Xu L., Zhang J., Yin L., Long X., Zhang W., Zhang Q. (2020). Recent progress in efficient organic two-photon dyes for fluorescence imaging and photodynamic therapy. J. Mater. Chem. C.

[27] Yang P.-P., Yang Y., Gao Y.-J., Wang Y., Zhang J.-C., Lin Y.-X., Dai L., Li J., Wang L., Wang H. (2015). Unprecedentedly High Tissue Penetration Capability of Co-Assembled Nanosystems for Two-Photon Fluorescence Imaging In Vivo. Adv. Opt. Mater..

[28] Hong Y., Lam J.W.Y., Tang B.Z. (2009). Aggregation-induced emission: Phenomenon, mechanism and applications. Chem. Commun..

[29] Wang Y., Han X., Xi W., Li J., Roe A.W., Lu P., Qian J. (2017). Bright AIE Nanoparticles with F127 Encapsulation for Deep-Tissue Three-Photon Intravital Brain Angiography. Adv. Healthc. Mater..

[30] Samanta S., Huang M., Li S., Yang Z., He Y., Gu Z., Zhang J., Zhang D., Liu L., Qu J. (2021). AIE-active two-photon fluorescent nanoprobe with NIR-II light excitability for highly efficient deep brain vasculature imaging. Theranostics.

[31] Shaw P.A., Forsyth E., Haseeb F., Yang S., Bradley M., Klausen M. (2022). Two-Photon Absorption: An Open Door to the NIR-II Biological Window?. Front. Chem..

[32] Chowdhury P., Chan Y.-H. (2022). Recent advances in D–A–D based Pdots with NIR-II fluorescence for deep-tissue imaging. Mol. Syst. Des. Eng..

[33] Qi J., Sun C., Li D., Zhang H., Yu W., Zebibula A., Lam J.W.Y., Xi W., Zhu L., Cai F. (2018). Aggregation-Induced Emission Luminogen with Near-Infrared-II Excitation and Near-Infrared-I Emission for Ultradeep Intravital Two-Photon Microscopy. ACS Nano.

[34] Hu W., Guo L., Bai L., Miao X., Ni Y., Wang Q., Zhao H., Xie M., Li L., Lu X. (2018). Maximizing Aggregation of Organic Fluorophores to Prolong Fluorescence Lifetime for Two-Photon Fluorescence Lifetime Imaging. Adv. Healthc. Mater..

[35] Wu W.-C., Chen C.-Y., Tian Y., Jang S.-H., Hong Y., Liu Y., Hu R., Tang B.Z., Lee Y.-T., Chen C.-T. (2010). Enhancement of Aggregation-Induced Emission in Dye-Encapsulating Polymeric Micelles for Bioimaging. Adv. Funct. Mater..

[36] Geng J., Goh C.C., Qin W., Liu R., Tomczak N., Ng L.G., Tang B.Z., Liu B. (2015). Silica shelled and block copolymer encapsulated red-emissive AIE nanoparticles with 50% quantum yield for two-photon excited vascular imaging. Chem. Commun..

[37] Geng J., Li K., Qin W., Ma L., Gurzadyan G.G., Tang B.Z., Liu B. (2013). Eccentric Loading of Fluorogen with Aggregation-Induced Emission in PLGA Matrix Increases Nanoparticle Fluorescence Quantum Yield for Targeted Cellular Imaging. Small.

[38] Alifu N., Sun Z., Zebibula A., Zhu Z., Zhao X., Wu C., Wang Y., Qian J. (2017). Deep-red polymer dots with bright two-photon fluorescence and high biocompatibility for in vivo mouse brain imaging. Opt. Commun..

[39] Khalin I., Heimburger D., Melnychuk N., Collot M., Groschup B., Hellal F., Reisch A., Plesnila N., Klymchenko A.S. (2020). Ultrabright Fluorescent Polymeric Nanoparticles with a Stealth Pluronic Shell for Live Tracking in the Mouse Brain. ACS Nano.

[40] Chuan-Xi W., Zhi-Yue G., Xin W., Can K., Zhuo Z., Chao-Jie Z., Li-Min F., Yuan W., Jian-Ping Z. (2019). Noninvasive and real-time pharmacokinetics imaging of polymeric nanoagents in the thoracoepigastric vein networks of living mice. J. Biomed. Opt..

[41] Liu J., Evrard M., Cai X., Feng G., Tomczak N., Ng L.G., Liu B. (2018). Organic nanoparticles with ultrahigh quantum yield and aggregation-induced emission characteristics for cellular imaging and real-time two-photon lung vasculature imaging. J. Mater. Chem. B.

[42] Shen F.-F., Chen Y., Xu X., Yu H.-J., Wang H., Liu Y. (2021). Supramolecular Assembly with Near-Infrared Emission for Two-Photon Mitochondrial Targeted Imaging. Small.

[43] Geng J., Li K., Ding D., Zhang X., Qin W., Liu J., Tang B.Z., Liu B. (2012). Lipid-PEG-Folate Encapsulated Nanoparticles with Aggregation Induced Emission Characteristics: Cellular Uptake Mechanism and Two-Photon Fluorescence Imaging. Small.

[44] Xiao X., Cai H., Huang Q., Wang B., Wang X., Luo Q., Li Y., Zhang H., Gong Q., Ma X. (2023). Polymeric dual-modal imaging nanoprobe with two-photon aggregation-induced emission for fluorescence imaging and gadolinium-chelation for magnetic resonance imaging. Bioact. Mater..

[45] Li S., Jiang X.-F., Xu Q.-H. (2018). Conjugated Polymers for Two-Photon Live Cell Imaging. Conjugated Polymers for Biological and Biomedical Applications.

[46] Wu C., Szymanski C., Cain Z., McNeill J. (2007). Conjugated Polymer Dots for Multiphoton Fluorescence Imaging. J. Am. Chem. Soc..

[47] Liu P., Li S., Jin Y., Qian L., Gao N., Yao S.Q., Huang F., Xu Q.-H., Cao Y. (2015). Red-Emitting DPSB-Based Conjugated Polymer Nanoparticles with High Two-Photon Brightness for Cell Membrane Imaging. ACS Appl. Mater. Interfaces.

[48] Goodwin A.P., Mynar J.L., Ma Y., Fleming G.R., Fréchet J.M.J. (2005). Synthetic Micelle Sensitive to IR Light via a Two-Photon Process. J. Am. Chem. Soc..

[49] Babin J., Pelletier M., Lepage M., Allard J.-F., Morris D., Zhao Y. (2009). A New Two-Photon-Sensitive Block Copolymer Nanocarrier. Angew. Chem. Int. Ed..

[50] Yuan Y., Wang Z., Cai P., Liu J., Liao L.-D., Hong M., Chen X., Thakor N., Liu B. (2015). Conjugated polymer and drug co-encapsulated nanoparticles for Chemo- and Photo-thermal Combination Therapy with two-photon regulated fast drug release. Nanoscale.

[51] Sun L., Yang Y., Dong C.-M., Wei Y. (2011). Two-Photon-Sensitive and Sugar-Targeted Nanocarriers from Degradable and Dendritic Amphiphiles. Small.

[52] Härtner S., Kim H.-C., Hampp N. (2007). Photodimerized 7-hydroxycoumarin with improved solubility in PMMA: Single-photon and two-photon-induced photocleavage in solution and PMMA films. J. Photochem. Photobiol. A Chem..

[53] Härtner S., Kim H.-C., Hampp N. (2007). Phototriggered release of photolabile drugs via two-photon absorption-induced cleavage of polymer-bound dicoumarin. J. Polym. Sci. Part A Polym. Chem..

[54] Kim H.-C., Härtner S., Behe M., Behr T., Hampp N. (2006). Two-photon absorption-controlled multidose drug release: A novel approach for secondary cataract treatment. J. Biomed. Opt..

[55] Ji W., Li N., Chen D., Qi X., Sha W., Jiao Y., Xu Q., Lu J. (2013). Coumarin-containing photo-responsive nanocomposites for NIR light-triggered controlled drug release via a two-photon process. J. Mater. Chem. B.

[56] Kehrloesser D., Behrendt P.J., Hampp N. (2012). Two-photon absorption triggered drug delivery from a polymer for intraocular lenses in presence of an UV-absorber. J. Photochem. Photobiol. A Chem..

[57] Huang Y., Shen L., Guo D., Yasen W., Wu Y., Su Y., Chen D., Qiu F., Yan D., Zhu X. (2019). A NIR-triggered gatekeeper of supramolecular conjugated unimicelles with two-photon absorption for controlled drug release. Chem. Commun..

[58] Olejniczak J., Sankaranarayanan J., Viger M.L., Almutairi A. (2013). Highest Efficiency Two-Photon Degradable Copolymer for Remote Controlled Release. ACS Macro Lett..

[59] Ma L.-L., Liu M.-X., Liu X.-Y., Sun W., Lu Z.-L., Gao Y.-G., He L. (2020). Macrocyclic polyamine [12]aneN3 modified triphenylamine-pyrazine derivatives as efficient non-viral gene vectors with AIE and two-photon imaging properties. J. Mater. Chem. B.

[60] Pack D.W., Hoffman A.S., Pun S., Stayton P.S. (2005). Design and development of polymers for gene delivery. Nat. Rev. Drug Discov..

[61] Wei L., Zhang D., Zheng X., Zeng X., Zeng Y., Shi X., Su X., Xiao L. (2018). Fabrication of Positively Charged Fluorescent Polymer Nanoparticles for Cell Imaging and Gene Delivery. Nanotheranostics.

[62] Zhao H., Tao H., Hu W., Miao X., Tang Y., He T., Li J., Wang Q., Guo L., Lu X. (2019). Two-Photon-Induced Charge-Variable Conjugated Polyelectrolyte Brushes for Effective Gene Silencing. ACS Appl. Bio Mater..

[63] Hayek A., Ercelen S., Zhang X., Bolze F., Nicoud J.-F., Schaub E., Baldeck P.L., Mély Y. (2007). Conjugation of a New Two-Photon Fluorophore to Poly(ethylenimine) for Gene Delivery Imaging. Bioconjugate Chem..

[64] Liu G., Xie J., Zhang F., Wang Z., Luo K., Zhu L., Quan Q., Niu G., Lee S., Ai H. (2011). N-Alkyl-PEI-Functionalized Iron Oxide Nanoclusters for Efficient siRNA Delivery. Small.

[65] Luo L., Zhang Q., Luo Y., He Z., Tian X., Battaglia G. (2019). Thermosensitive nanocomposite gel for intra-tumoral two-photon photodynamic therapy. J. Control. Release.

[66] Luo L., Yin Z., Qi Y., Liu S., Yi Y., Tian X., Wu Y., Zhong D., Gu Z., Zhang H. (2021). An intracellular enzyme-responsive polymeric prodrug with synergistic effect of chemotherapy and two-photon photodynamic therapy. Appl. Mater. Today.

[67] Kandoth N., Kirejev V., Monti S., Gref R., Ericson M.B., Sortino S. (2014). Two-Photon Fluorescence Imaging and Bimodal Phototherapy of Epidermal Cancer Cells with Biocompatible Self-Assembled Polymer Nanoparticles. Biomacromolecules.

[68] Wang H., Di J., Sun Y., Fu J., Wei Z., Matsui H., del C. (2015). Alonso, A.; Zhou, S. Biocompatible PEG-Chitosan@Carbon Dots Hybrid Nanogels for Two-Photon Fluorescence Imaging, Near-Infrared Light/pH Dual-Responsive Drug Carrier, and Synergistic Therapy. Adv. Funct. Mater..

[69] Ardekani S.M., Dehghani A., Hassan M., Kianinia M., Aharonovich I., Gomes V.G. (2017). Two-photon excitation triggers combined chemo-photothermal therapy via doped carbon nanohybrid dots for effective breast cancer treatment. Chem. Eng. J..

[70] Ma B., Zhuang W., Xu H., Li G., Wang Y. (2019). Hierarchical Responsive Nanoplatform with Two-Photon Aggregation-Induced Emission Imaging for Efficient Cancer Theranostics. ACS Appl. Mater. Interfaces.

[71] Xu H., Ma B., Jiang J., Xiao S., Peng R., Zhuang W., Li G., Wang Y. (2019). Integrated prodrug micelles with two-photon bioimaging and pH-triggered drug delivery for cancer theranostics. Regen. Biomater..

[72] Yu T., Zhuang W., Su X., Ma B., Hu J., He H., Li G., Wang Y. (2019). Dual-Responsive Micelles with Aggregation-Induced Emission Feature and Two-Photon Aborsption for Accurate Drug Delivery and Bioimaging. Bioconjugate Chem..

[73] He H., Zhuang W., Ma B., Su X., Yu T., Hu J., Chen L., Peng R., Li G., Wang Y. (2019). Oxidation-Responsive and Aggregation-Induced Emission Polymeric Micelles with Two-Photon Excitation for Cancer Therapy and Bioimaging. ACS Biomater. Sci. Eng..

[74] Zhuang W., Ma B., Hu J., Jiang J., Li G., Yang L., Wang Y. (2019). Two-photon AIE luminogen labeled multifunctional polymeric micelles for theranostics. Theranostics.

[75] Ma B., Xu H., Zhuang W., Wang Y., Li G., Wang Y. (2020). ROS Responsive Nanoplatform with Two-Photon AIE Imaging for Atherosclerosis Diagnosis and “Two-Pronged” Therapy. Small.

[76] Kirejev V., Kandoth N., Gref R., Ericson M.B., Sortino S. (2014). A polymer-based nanodevice for the photoregulated release of NO with two-photon fluorescence reporting in skin carcinoma cells. J. Mater. Chem. B.

